# Longitudinal growth and emotional and behavioral problems at age 7 in moderate and late preterms

**DOI:** 10.1371/journal.pone.0211427

**Published:** 2019-01-31

**Authors:** Baukje M. Dotinga, Andrea F. de Winter, Inger F. A. Bocca-Tjeertes, Jorien M. Kerstjens, Sijmen A. Reijneveld, Arend F. Bos

**Affiliations:** 1 Division of Neonatology, Department of Pediatrics, Beatrix Children’s Hospital, University Medical Center Groningen, University of Groningen, Groningen, The Netherlands; 2 Department of Health Sciences, University Medical Center Groningen, University of Groningen, Groningen, The Netherlands; Utrecht University, NETHERLANDS

## Abstract

**Objectives:**

Moderately and late preterm children (MLPs, 32.0–36.9 weeks gestational age) have a greater risk of poorer growth. This seems to be associated with poorer neuropsychological functioning. Evidence is limited on whether this also holds for emotional and behavioral (EB) problems. Therefore, we assessed whether longitudinal growth from birth until age 7 was associated with EB problems at age 7 in MLPs.

**Study design:**

This study was part of the Longitudinal Preterm Outcome Project, a prospective cohort study. Data on growth (height, weight, head circumference, and extent of catch-up growth) were obtained from assessments from birth until age 7. EB problems were assessed at age 7 with the Child Behavior Checklist. We assessed whether growth and EB problems were associated using logistic regression analyses, adjusting for multiple birth, parity, and socioeconomic status.

**Results:**

We included 248 MLPs. Median gestational age was 34 weeks (interquartile range: 33–35 weeks). Mean birth weight was 2.2 kg (standard deviation: 0.5 kg). Postnatal growth measures were below the Dutch reference norm. EB problems were more prevalent in MLPs than in the general Dutch population. Generally, we found no associations between growth and EB problems; odds ratios ranged from 0.20 to 2.72.

**Conclusions:**

In MLPs, postnatal growth from birth until age 7 was not associated with EB problems at age 7. Poorer growth thus seems to relate to neuropsychological problems, but not to EB problems. This suggests that the etiologies of these problems differ at least partially.

## Introduction

Worldwide, approximately 10% of all children are born preterm [1]. Moderate (32.0–33.9 weeks gestational age, GA) and late (34.0–36.9 weeks GA) prematurity accounts for approximately 80% of all preterm births [1]. Moderately and late preterm (MLP) birth leads to a greater risk of adverse childhood outcomes, including growth restraint [2], impaired neuropsychological functioning [3], and emotional and behavioral (EB) problems [4–8]. Previous studies have shown that in preterm-born children, these adverse outcomes co-occur and may be interrelated: poorer growth seems to be associated with poorer neuropsychological functioning [9,10] and children with poorer neuropsychological functioning often also have more EB problems [11,12]. In MLPs, EB problems mainly concern mood and emotion disturbances [8,13]. EB problems in childhood have been associated with a variety of mental health disorders in later life, including anxiety and depressive disorders [14–16]. Gaining more insight into the etiology of EB problems in childhood may improve detection, intervention strategies, and outcomes of these problems.

Poorer neuropsychological functioning and EB problems may result from disruptions in brain development [17–20]. Several studies indicate that brain development of preterm children can be permanently affected by nutrient restriction [21,22]. Therefore, poorer growth, as an indicator of nutrient restriction, may be associated with poorer neurodevelopmental outcomes. Numerous studies have already shown this for growth and neuropsychological functioning [9], but few studies have addressed this hypothesis regarding EB problems. Moreover, the results of these few studies are not in line with each other. Some studies described an inverse association between growth and EB problems [23,24], i.e. poorer growth was associated with more EB problems. Other studies, however, were not able to demonstrate any association [25–28]. Furthermore, data on postnatal growth is limited, even though brain maturation processes including synaptogenesis and myelination continue throughout childhood and adolescence [29,30]. Finally, evidence lacks on MLPs specifically. Nevertheless, MLPs are born during a period of important brain growth and maturation [31]. During this rapid growth, the brain seems to be especially vulnerable to postnatal nutrient restriction [21,22]. Therefore, we aimed to determine whether longitudinal growth from birth until the age of 7 years is associated with EB problems at the age of 7 years in MLPs.

## Methods

### Patients

This study was part of the Longitudinal Preterm Outcome Project (LOLLIPOP, registered at controlled-trials.com: ISRCTN80622320), a prospective cohort study that was designed to investigate growth, development, and general health of preterm children, with emphasis on MLP birth. The study was approved by the Ethical Review Board of the University Medical Center of Groningen.

Children were included during routine well-child care in preventive child health care organizations at the age of 4 years. These organizations assess more than 90% of the children in The Netherlands at regular intervals [32]. Children were enrolled between January 2002 and June 2003. At the time the study was designed, the term “moderate prematurity” was defined as birth at 32.0–35.9 weeks GA. Therefore, children born at 36.0–36.9 weeks GA were not included. Major congenital malformations and chromosomal abnormalities or syndromes were exclusion criteria. Sampling procedures, inclusion and exclusion criteria, study conduct, participants, and non-participants in the LOLLIPOP study were previously described in more detail [33].

From this community-based cohort, we invited parents of all 341 MLPs from the three northern provinces in the Netherlands to fill in questionnaires when their child had reached the age of 7 years. In total, 248 parents (73%) participated.

### Measures and procedures

#### Growth

Data on growth were obtained from records on well-child assessments from birth to the age of 4 years and from data collected additionally in a research setting at the age of 7 years. In The Netherlands, children have approximately 15 routine well-child assessments in preventive child health care organizations to monitor their mental and physical development, using structured interviews with parents, general physical examinations, and standardized screening procedures. Head circumference (HC) was assessed until the large fontanel was closed. Height and weight were measured using standardized measuring devices, i.e. an infantometer or stadiometer. Children were examined in the supine position until they reached the age of 24 months. From 24 months onwards the child was standing during measurements. We only present data on growth at birth and at the ages of 1, 4 and 7 years, because growth measures at these ages were available in most children.

We converted all growth measures to z-scores based on the best available norm data. Height and HC at birth were converted according to Niklasson [34]. Weight at birth was converted using the Dutch Kloosterman curve and classified as small for gestational age for the lowest 10 percent and as large for gestational age for the highest 10 percent [35]. Growth measures at the ages of 1, 4 and 7 years were converted using the fourth Dutch nationwide growth study [36]. We measured catch-up growth as changes in consecutive z-scores. For the postnatal growth measures, we did not correct for prematurity, as it is convention not to correct after the age of 24 months. To understand and interpret changes in the postnatal z-scores more easily, using the same growth charts, we chose to refrain from correction regarding the 1 year measurements as well.

#### EB problems

We measured EB problems at the age of 7 years using the validated Dutch version of the Child Behavior Checklist (CBCL) for 6–18 years [37,38]. The CBCL has good psychometric properties and is often considered the gold standard for behavior rating scales [37]. It consists of 112 parent-reported problem items and an open-ended item on any problems that were not listed on the form. Each of the problems items could be scored as: not true (0), somewhat/sometimes true (1), or very/often true (2). We constructed total, internalizing and externalizing problem scales by summing the scores for sets of items. Internalizing problems are characterized by disordered mood or emotion, e.g. withdrawal and anxiety, whereas externalizing problems are characterized by disinhibited behavior, e.g. aggression [39]. We classified these problem scales as normal (<84th centile of the US norm population), subclinical (≥84th and <90th centile) or clinical (≥90th centile) [37].

The Strengths and Difficulties Questionnaire (SDQ) is a brief behavioral screening questionnaire that is more widely used in clinical settings, because of its brevity and its ability to measure competences as well as problem behaviors [40,41]. Moreover, the SDQ seems to be better than the CBCL at detecting inattention and hyperactivity [42]. Therefore, we also used the hyperactivity subscale and the total difficulties score from the Dutch version of the SDQ. We classified these scores as normal, borderline or abnormal according to Dutch norms [43].

#### Covariates

We selected covariates based on previous studies on EB problems in preterm children [44–47]. Perinatal characteristics included gender, small-for-gestational age at birth and being part of a multiple pregnancy. Family characteristics included parity of the mother and socioeconomic status (SES). We computed a composite SES measure using standardized scores on five indicators: educational level of both parents, family income and occupational level of both parents [48,49]. Educational level was categorized as: (1) primary school or less, (2) low-level technical and vocational training (<12 years’ education), (3) high school or medium-level technical and vocational training (12–16 years’ education), and (4) university or high-level technical and vocational training (>16 years’ education). Occupational level was classified according to the International Standard Classification of Occupations [50]. Next, we categorized as: (1) low SES, i.e. scores more than 1 SD below the mean; (2) intermediate SES, i.e. scores between 1 SD below the mean and 1 SD greater than the mean; and (3) high SES, i.e. scores more than 1 SD greater than the mean.

### Data and statistical analyses

First, we assessed background characteristics of the sample. Second, we assessed prevalence rates of clinical and subclinical EB problems. Third, we assessed whether growth (i.e. attained height, weight, and HC and gain in height, weight, and HC) and clinical EB problems were associated using univariable and multivariable logistic regression analyses. The multivariable analyses were corrected for multiple birth, parity and SES. To assess the effect of gain in height, weight, and HC between two consecutive time points, we also corrected those analyses for height, weight, and HC of the first time point, respectively. We repeated the logistic regression analyses for the total difficulties score and the hyperactivity subscale of the SDQ.

We used IBM SPSS version 23.0 (IBM Corp, Armonk, NY) for statistical analyses. Correcting for multiple comparisons (Bonferroni), we considered a *P*-value of less than 0.01 to be statistically significant.

## Results

We included 248 MLPs, with a median GA of 34 weeks (interquartile rage: 33–35 weeks) and a mean birth weight of 2.2 kg (SD: 0.5 kg). Background characteristics are shown in Table 1. Compared with the participating children, fewer non-participating children were small-for-gestational age at birth and more came from families with low SES. Other characteristics, including gender, gestational age and birth weight, did not differ significantly between the two groups.

**Table 1:** Characteristics of the sample of moderately and late preterm children.

		Participants (n = 248)	Non-participants (n = 93)
Gender (male), n (%)		138 (55.6)	60 (64.5)
Gestational age (weeks), median (IQR)		34 (33–35)	34 (34–35)
Birth weight (kg), mean (SD)		2.2 (0.5)	2.3 (0.4)
Small for gestational age, n (%)	total	31 (12.5)	3 (3.2)*
	singletons	24 (12.8)	
	multiples	7 (11.6)	
Large for gestational age, n (%)		31 (12.5)	12 (12.9)
Multiples, n (%)	total	60 (24.6)	21 (22.6)
	twins	58 (96.7)	
	triplets and quadruplets	2 (3.3)	
Parity (multiparae), n (%)		81 (32.7)	35 (37.6)
SES ^a^, n (%)	low	65 (26.2)	41 (44.1)*
	intermediate	129 (52.0)	41 (44.1)
	high	51 (20.6)	10 (10.8)*

IQR: interquartile range. SD: standard deviation

^a^ Low SES, scores ≤ mean—1 SD on standardized SES scale; intermediate SES, scores > mean—1 SD and ≤ mean + 1 SD; high SES, scores > mean + 1 SD

* *P* < 0.05

In Table 2, we present height, weight, HC, and corresponding z-scores at birth and the ages of 1, 4, and 7 years as well as data on growth, i.e. changes in consecutive z-scores. The MLPs were smaller than the norm for all postnatal growth measures. However, both height gain and weight gain between the ages of 1 and 4 years showed a positive change in z-scores, indicating that catch-up growth occurred to some extent.

**Table 2:** Growth outcomes of the sample of moderately and late preterm children, presented as raw scores and z-scores.

		raw score, mean (SD)	z-score, mean (SD)
Height (cm)	0 yr	45.2 (3.3)	-0.270 (2.1)
	1 yr	74.1 (2.6)	-0.443 (1.4)
	4 yr	103.7 (4.0)	-0.123 (1.0)
	7 yr	124.8 (5.4)	-0.212 (1.0)
Height gain (cm)	0–1 yr	29.0 (3.2)	-0.110 (2.0)
	1–4 yr	29.5 (2.9)	0.380 (0.7)
	4–7 yr	21.1 (2.7)	-0.080 (0.5)
Weight (kg)	0 yr	2.2 (0.5)	0.105 (1.0)
	1 yr	9.4 (1.1)	-0.387 (1.4)
	4 yr	16.6 (2.3)	-0.203 (1.0)
	7 yr	24.4 (4.8)	-0.116 (1.3)
Weight gain (kg)	0–1 yr	7.1 (0.9)	-0.560 (1.0)
	1–4 yr	7.3 (1.9)	0.210 (0.8)
	4–7 yr	7.8 (2.9)	0.100 (0.7)
HC (cm)	0 yr	31.4 (1.8)	-0.190 (1.1)
	1 yr	46.5 (1.4)	-0.044 (0.9)
HC gain (cm)	0–1 yr	14.9 (1.7)	0.040 (1.0)

**Fig 1:**
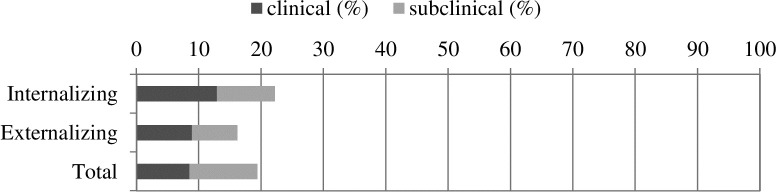
Prevalence rates of emotional and behavioral problems in moderately and late preterm children. ‘Clinical’ indicates scores ≥90th centile and ‘subclinical’ indicates scores ≥84th centile and <90th centile. In The Netherlands, prevalence rates of clinical problems are approximately 8%, 5% and 6% for internalizing, externalizing and total problem scales, respectively [51,52].

Fig 1 shows prevalence rates for clinical and subclinical EB problems. Among the MLPs, 12.9% had an internalizing score in the clinical range and 9.3% in the subclinical range (combined 22.2%). For externalizing problems, 8.9% had a score in the clinical range and 7.3% in the subclinical range (combined 16.2%). For total problems 8.5% had score in the clinical range and 10.9% in the subclinical range (combined 19.4%).

We found no associations between growth from birth until the age of 7 years and EB problems at the age of 7 years, with the exception of height gain (Table 3). Greater gain in height from birth to the age of 1 year increased the risk of internalizing problems at the age of 7 years (odds ratio 2.72, 95% confidence interval 1.40–5.30). Since the association was only significant in the multivariable analysis, we repeated the analysis adjusting for only one variable at a time. Then, the association was only significant after adjusting for parity (odds ratio 2.73, 95% confidence interval 1.41–5.26). The logistic regression analyses for SDQ outcomes yielded similar results; odds ratios ranged from 0.47 to 3.47 (S1 Table).

**Table 3:** Risk of clinical emotional and behavioral problems by growth, adjusted for multiple birth, parity, and socioeconomic status: Odds ratios with 95% confidence intervals.

			Internalizing		Externalizing		Total score	
			Univariable	Multivariable	Univariable	Multivariable	Univariable	Multivariable
Height	0 yr	*n = 189*	0.90 (0.74–1.09)	0.79 (0.63–0.99)	0.98 (0.78–1.24)	0.88 (0.66–1.16)	0.91 (0.72–1.14)	0.78 (0.59–1.03)
	1 yr	*n = 158*	1.14 (0.88–1.46)	1.51 (0.93–2.45)	1.08 (0.79–1.48)	1.56 (0.80–3.06)	1.06 (0.78–1.45)	1.31 (0.74–2.34)
	4 yr	*n = 210*	1.36 (0.89–2.06)	1.45 (0.91–2.32)	1.68 (1.02–2.75)	1.64 (0.93–2.91)	1.42 (0.88–2.29)	1.48 (0.86–2.54)
	7 yr	*n = 210*	1.13 (0.76–1.67)	1.13 (0.73–1.75)	1.40 (0.89–2.21)	1.26 (0.74–2.17)	1.38 (0.88–2.18)	1.46 (0.86–2.49)
Height gain^#^	0–1 yr	*n = 130*	1.19 (0.96–1.46)	2.72 (1.40–5.30)*	1.13 (0.87–1.48)	2.50 (1.10–5.67)	1.22 (0.96–1.57)	2.55 (1.17–5.58)
	1–4 yr	*n = 141*	0.94 (0.51–1.76)	1.25 (0.61–2.57)	1.50 (0.67–3.36)	2.20 (0.73–6.66)	1.46 (0.70–3.06)	1.74 (0.71–4.22)
	4–7 yr	*n = 191*	0.58 (0.24–1.40)	0.62 (0.24–1.64)	0.52 (0.19–1.41)	0.47 (0.15–1.55)	0.69 (0.26–1.78)	0.85 (0.29–2.48)
Weight	0 yr	*n = 189*	0.85 (0.56–1.27)	0.77 (0.51–1.16)	1.24 (0.79–1.95)	1.18 (0.73–1.90)	0.82 (0.50–1.35)	0.75 (0.46–1.22)
	1 yr	*n = 158*	1.06 (0.81–1.39)	1.12 (0.72–1.72)	1.06 (0.75–1.49)	1.14 (0.65–2.00)	0.93 (0.61–1.42)	0.86 (0.50–1.47)
	4 yr	*n = 210*	1.20 (0.82–1.75)	1.14 (0.76–1.73)	1.33 (0.88–2.03)	1.15 (0.70–1.89)	1.03 (0.66–1.60)	0.95 (0.58–1.56)
	7 yr	*n = 210*	0.92 (0.66–1.27)	0.81 (0.55–1.19)	1.09 (0.77–1.54)	0.85 (0.53–1.35)	1.00 (0.70–1.44)	0.86 (0.55–1.33)
Weight gain^#^	0–1 yr	*n = 130*	1.32 (0.84–2.08)	1.33 (0.80–2.23)	0.99 (0.55–1.78)	1.13 (0.59–2.19)	1.26 (0.71–2.22)	1.07 (0.58–2.00)
	1–4 yr	*n = 141*	1.14 (0.67–1.94)	1.08 (0.62–1.88)	1.21 (0.62–2.37)	1.23 (0.58–2.62)	1.29 (0.71–2.37)	1.22 (0.65–2.31)
	4–7 yr	*n = 191*	0.62 (0.35–1.12)	0.59 (0.32–1.09)	0.68 (0.37–1.25)	0.20 (0.05–0.83)	0.78 (0.42–1.45)	0.58 (0.28–1.19)
HC	0 yr	*n = 167*	1.18 (0.80–1.74)	1.05 (0.70–1.58)	1.43 (0.81–2.51)	1.19 (0.66–2.14)	0.85 (0.53–1.36)	0.72 (0.43–1.19)
	1 yr	*n = 172*	1.23 (0.76–2.02)	1.23 (0.70–2.15)	0.87 (0.46–1.63)	0.81 (0.39–1.70)	0.89 (0.47–1.66)	0.78 (0.38–1.62)
HC gain^#^	0–1 yr	*n = 129*	0.88 (0.56–1.38)	0.71 (0.36–1.39)	1.62 (0.74–3.56)	1.81 (0.56–5.85)	0.72 (0.41–1.29)	0.72 (0.39–1.32)

^#^ The multivariable analyses for gain in height, weight, and HC between two time points were also corrected for height, weight, and HC of the first time point, respectively.

* *P* < 0.01.

## Discussion

This study showed that nearly all measures of longitudinal growth from birth until the age of 7 years were not associated with EB problems at the age of 7 years in MLPs. MLPs only had a greater risk of internalizing problems at the age of 7 years in case of greater height gain in the first year of life.

These results do not support our hypothesis that poorer growth is associated with EB problems. This contrasts with findings of Pyhälä et al. who reported an association between poorer growth from birth to term age and higher levels of autism-spectrum traits in adolescence for children born with very low birth weight (<1,500 grams) [23]. We were unable to confirm those findings in an MLP population, as the CBCL does not focus on those traits. Moreover, our findings contrast with several studies that described an association between birth weight and EB problems [24,53]. We may not have found that association, due to limited diversity in birth weight: only 16 infants (6.5%) had a birth weight below 1,500 grams.

Our results are consistent with those of previous studies that were unable to demonstrate an association between growth and EB problems [25–28]. However, none of these studies addressed this in MLPs only; they included all preterm children or had inclusion criteria based on birth weight. A recent study by Huang et al. showed no associations between growth and childhood EB problems in a Chinese cohort of 654 preterm children [27]. However, they did not assess postnatal height and HC and they measured attained weight at a single time point between the ages of 4–7 years. We extend their findings by showing that neither postnatal height and HC gain, nor postnatal weight gain during specific postnatal periods are associated with EB problems in childhood.

In our study, height gain in the first year of life was the only growth measure that was associated with EB problems. To our knowledge, we are the first to report a positive association between growth and EB problems. Since only a single association came out significantly and only in the multivariable analysis, we believe this may be a chance finding.

An important strength of this study is its community-based sample. Furthermore, our analyses were comprehensively adjusted for effects that can be attributed to SES, as we used a composite score of the three most often used indicators of SES, i.e. education, income and occupation [48]. Our study also has some limitations to address. First, our study sample size was relatively small and did not include children born between 36.0–36.9 weeks GA. A larger sample may have yielded similar results, though, given that the odds ratios in our study ranged from 0.20 to 2.72. Second, we assessed EB problems using parental reports of symptoms rather than obtaining a DSM diagnosis. However, both the CBCL and the SDQ are very valid and widely used instruments [37,41].

Our findings imply that growth has hardly any effect on childhood EB problems. It seems that growth is associated with both neuropsychological functioning [[9,10]] and autism-spectrum traits [23], but not with EB problems. Preterm birth increases the risk of all these problems, yet the lesions may be specific for each problem and differ in terms of timing and reversibility. This could explain why growth seems to affect only specific domains. Moreover, the role of factors other than preterm birth and growth may be more important for developing EB problems. These may include social and environmental factors, such as low socio-economic status [47], lower maternal age at birth [45,54], maternal depression [55], and living in a single-parent family [54,56]. Future research is needed to gain more insight in the etiologies of these problems and to identify contributing factors.

## Conclusion

In MLPs, longitudinal growth from birth until the age of 7 years was not associated with EB problems at the age of 7 years. These findings suggest that the etiologies of EB problems and of poorer neuropsychological functioning are at least partly different. These etiologies require further research.

## Supporting information

S1 Table Risk of clinical emotional and behavioral problems by growth, adjusted for SES, multiple birth and multiparity: Odds ratios with 95% confidence intervals. # The multivariable analyses for gain in height, weight, and HC between two time points were also corrected for height, weight, and HC of the first time point, respectively. * P < 0.01.
(DOCX)
