# Supplementary material for: Longitudinal growth and emotional and behavioral problems at age 7 in moderate and late preterms
Source: PLoS One. 2019 Jan 31;14(1):e0211427. doi: 10.1371/journal.pone.0211427 (PMC6355004; doi:10.1371/journal.pone.0211427)
Supplement: S1 Table — # The multivariable analyses for gain in height, weight, and HC between two time points were also corrected for height, weight, and HC of the first time point, respectively. * P < 0.01. (DOCX) [file pone.0211427.s001.docx]

**S1 Table. Risk of clinical emotional and behavioral problems by growth, adjusted for SES, multiple birth and multiparity: odds ratios with 95% confidence intervals**

|  |  |  | Hyperactivitiy | | Total difficulties | |  |
| --- | --- | --- | --- | --- | --- | --- | --- |
|  |  |  | Univariable | Multivariable | Univariable | Multivariable | Multivariable |
| Height | 0 yr | *n = 189* | 0.87 (0.71-1.07) | 0.76 (0.60-0.97) | 0.89 (0.73-1.07) | 0.83 (0.66-1.04) |  |
|  | 1 yr | *n = 158* | 0.87 (0.54-1.39) | 0.96 (0.56-1.65) | 1.10 (0.85-1.43) | 1.44 (0.88-2.37) |  |
|  | 4 yr | *n = 210* | 0.91 (0.60-1.37) | 0.95 (0.60-1.51) | 1.61 (1.02-2.54) | 1.83 (1.08-3.07) |  |
|  | 7 yr | *n = 210* | 0.93 (0.61-1.40) | 0.96 (0.60-1.53) | 1.27 (0.84-1.92) | 1.21 (0.76-1.93) |  |
| Height gain^#^ | 0-1 yr | *n = 130* | 1.13 (0.89-1.44) | 1.47 (0.77-2.78) | 1.18 (0.95-1.46) | 2.06 (1.12-3.77) |  |
|  | 1-4 yr | *n = 141* | 1.20 (0.57-2.50) | 1.27 (0.55-2.92) | 1.55 (0.77-3.12) | 2.04 (0.88-4.73) |  |
|  | 4-7 yr | *n = 191* | 1.07 (0.45-2.54) | 1.10 (0.41-2.99) | 0.67 (0.26-1.71) | 0.62 (0.21-1.85) |  |
| Weight | 0 yr | *n = 226* | 0.72 (0.47-1.11) | 0.62 (0.39-0.99) | 0.91 (0.60-1.36) | 0.85 (0.56-1.29) |  |
|  | 1 yr | *n = 158* | 0.83 (0.52-1.32) | 0.89 (0.53-1.49) | 1.04 (0.77-1.40) | 1.10 (0.70-1.73) |  |
|  | 4 yr | *n = 210* | 0.92 (0.61-1.38) | 0.95 (0.60-1.51) | 1.46 (0.99-2.16) | 1.48 (0.97-2.28) |  |
|  | 7 yr | *n = 208* | 0.80 (0.56-1.15) | 0.73 (0.49-1.10) | 1.07 (0.77-1.47) | 0.96 (0.66-1.39) |  |
| Weight gain^#^ | 0-1 yr | *n = 146* | 1.22 (0.71-2.09) | 1.06 (0.60-1.90) | 1.26 (0.78-2.02) | 1.26 (0.74-2.13) |  |
|  | 1-4 yr | *n = 141* | 1.36 (0.75-2.48) | 1.35 (0.70-2.60) | 1.61 (0.91-2.88) | 1.67 (0.92-3.03) |  |
|  | 4-7 yr | *n = 190* | 0.74 (0.41-1.32) | 0.59 (0.29-1.20) | 0.77 (0.42-1.41) | 0.53 (0.26-1.07) |  |
| HC | 0 yr | *n = 167* | 0.94 (0.62-1.41) | 0.73 (0.45-1.17) | 1.29 (0.85-1.98) | 1.08 (0.68-1.69) |  |
|  | 1 yr | *n = 172* | 0.49 (0.24-1.01) | 0.47 (0.21-1.05) | 1.00 (0.59-1.71) | 0.95 (0.52-1.73) |  |
| HC gain^#^ | 0-1 yr | *n = 129* | 1.71 (0.83-3.52) | 3.47 (1.08-11.18) | 1.79 (0.95-3.35) | 1.62 (0.69-3.84) |  |

^#^ The multivariable analyses for gain in height, weight, and HC between two time points were also corrected for height, weight, and HC of the first time point, respectively.

* *P* < 0.01
